# IKIP downregulates THBS1/FAK signaling to suppress migration and invasion by glioblastoma cells

**DOI:** 10.32604/or.2024.042456

**Published:** 2024-06-20

**Authors:** ZHAOYING ZHU, YANJIA HU, FENG YE, HAIBO TENG, GUOLIANG YOU, YUNHUI ZENG, MENG TIAN, JIANGUO XU, JIN LI, ZHIYONG LIU, HAO LIU, NIANDONG ZHENG

**Affiliations:** 1 Department of Neurosurgery, The Affiliated Hospital of Southwestern Medical University, Luzhou, China; 2 Department of Neurosurgery, Sichuan University West China Hospital, Chengdu, China

**Keywords:** Inhibitor of NF-κB kinase-interacting protein (IKIP), Glioblastoma (GBM), Migration, Thrombospondin 1 (THBS1), FAK signaling

## Abstract

**Background:**

Inhibitor of NF-κB kinase-interacting protein (IKIP) is known to promote proliferation of glioblastoma (GBM) cells, but how it affects migration and invasion by those cells is unclear.

**Methods:**

We compared levels of IKIP between glioma tissues and normal brain tissue in clinical samples and public databases. We examined the effects of IKIP overexpression and knockdown on the migration and invasion of GBM using transwell and wound healing assays, and we compared the transcriptomes under these different conditions to identify the molecular mechanisms involved.

**Results:**

Based on data from our clinical samples and from public databases, IKIP was overexpressed in GBM tumors, and its expression level correlated inversely with survival. IKIP overexpression in GBM cells inhibited migration and invasion in transwell and wound healing assays, whereas IKIP knockdown exerted the opposite effects. IKIP overexpression in GBM cells that were injected into mouse brain promoted tumor growth but inhibited tumor invasion of surrounding tissue. The effects of IKIP were associated with downregulation of THBS1 mRNA and concomitant inhibition of THBS1/FAK signaling.

**Conclusions:**

IKIP inhibits THBS1/FAK signaling to suppress migration and invasion of GBM cells.

## Introduction

Glioblastoma is associated with one of the highest mortality rates among cancers of the central nervous system [1]: even with aggressive treatment, the median survival is approximately 15 months [2]. Glioblastoma tumors are highly invasive, so they colonize adjacent brain tissues that cannot be excised safely, leading to recurrence [3]. Understanding what proteins and processes regulate migration and invasion of glioblastoma cells may improve treatments.

One potential regulator is inhibitor of NF-κB kinase-interacting protein (IKIP), which is upregulated in glioblastoma and which promotes proliferation of the tumor cells by suppressing degradation of CDK4, thereby driving progression through the cell cycle [4]. Transient knockdown of IKIP in GBM cell lines inhibits their proliferation, and its expression level in patients correlates inversely with survival [5]. While that study also suggested that IKIP promotes migration [5], the exact function and potential mechanism are not clear.

Here we confirm, in patient biopsies from our clinic and in public databases, that IKIP is upregulated in glioblastoma tumors and that its expression correlates inversely with prognosis. We further show, *in vitro* and *in vivo*, that although IKIP promotes proliferation of tumor cells, it suppresses their migration and invasion. We provide evidence that IKIP exerts its effects by downregulating THBS1/FAK signaling. These findings uncover a dual role of IKIP in the modulation of growth and migration of glioblastoma tumors.

## Materials and Methods

### Public data

Data on patient characteristics and gene expression in GBM tumors and normal brain tissue were downloaded from the Chinese Glioma Genome Atlas (www.cgga.org.cn), the Cancer Genome Atlas (http://tcgaportal.org) and the Rembrandt database.

### Clinical samples

Glioma tissues were obtained from patients treated at West China Hospital, Sichuan University (Chengdu, China). The use of human tissues was approved by the hospital’s Institutional Review Board, and patients provided written consent for their tissues and anonymized medical information to be analyzed and published for research purposes.

### Cell culture

The human embryonic kidney cell line HEK293T as well as the human glioblastoma cell lines U87-MG and LN229 were purchased from Meisen Cell (Zhejiang, China) and cultured in Dulbecco’s modified Eagle medium (DMEM) supplemented with 10% fetal bovine serum and 1% penicillin/streptomycin. Cells were cultured at 37°C in a humidified atmosphere containing 5% CO_2_. All cell lines were authenticated through analysis of short tandem repeats.

### Reagents and antibodies

RIPA lysis buffer (catalog no. P0013B) and enhanced BCA protein assay (P0010) were purchased from Beyotime (Shanghai, China), while HiScript II Q RT SuperMix for qPCR (catalog no. R222-01) and ChamQ Universal SYBR qPCR Master Mix (Q711-02) were purchased from Vazyme (Nanjing, China). Other major reagents included jetPRIME Versatile DNA/siRNA transfection reagent (catalog no. 101000046, Polyplus, Strasbourg, France), Matrigel® matrix (356234, Corning, USA), fetal bovine serum (BS1612-109, Bioexplorer, USA), penicillin/streptomycin (15070063, Gibco, USA), and Cell Counting kit-8 (M4839, Abmole, USA).

Primary antibodies in this study recognized the following proteins: β-tubulin (catalog no. SR25-04, Huabio, Hangzhou, China), FAK (ET1602-04, Huabio), phospho-FAK (SC54-07, Huabio; EP2160Y, Abcam, USA), fibronectin (ET1702-25, Huabio; ab258174, Abcam), paxillin (ab32084, Abcam), Ki67 (HA721115, Huabio), or IKIP (ab221171, Abcam; 14589-1-AP, ProteinTech, Wuhan, China).

The following secondary antibodies from goat that recognized rabbit IgG were used in the study: a horseradish peroxidase-conjugated anti-IgG (H+L) antibody (catalog no. S0001, Affinity, Changzhou, China) and polyclonal antibody conjugated to either iFluor™ 488 (HA1121, Huabio) or iFluor™ 594 (HA1122, Huabio).

### Overexpression and knockdown of IKIP in GBM cells

In certain experiments, glioblastoma cells were transduced with recombinant lentivirus encoding human IKIP, a short hairpin RNA targeting endogenous IKIP, or no transgene (empty vector). Recombinant viruses were prepared and produced in HEK293T cells by co-transfecting the lentiviral plasmid with the packaging plasmids pMD2.G and psPAX using jetPRIME® transfection reagent according to the manufacturer’s protocol.

In other experiments, GBM cells were generated lacking the gene encoding IKIP. The vector lentiCRISPRv2 (a gift from Prof. Yaohui Chen) was digested, dephosphorylated with *Bsm*BI endonuclease, and ligated with one of the following oligos (annealed to its complementary oligo) that had been designed in the CRISPR Design tool (http://crispr.mit.edu) to produce single-guide RNAs: sgIKIP-1, ACCCCCGTGGCCCGGAGCAG; and sgIKIP-2, GGTTTGTATTTCAGCAGTCA. Each lentiviral plasmid was transfected into HEK293T cells with packaging plasmids as above to produce recombinant lentivirus, which was used to infect LN229 and U87-MG cells. Knockdown of IKIP was confirmed by western blotting (see below).

### Knockdown of THBS1 in glioblastoma cells

Cell lines were transfected with one of the following two small interfering RNAs targeting THBS1 (Youkang Biotechnology, Hangzhou, China): siTHBS1-1, 5′-GCGTGTTTGACATCTTTGA-3′; and siTHBS1-2, 5′-CTGCGTTGGTGATGTAACA-3′. We used a non-silencing siRNA as a control: 5′-GUACCGCACGUCAUUCGUAUC-3′. The concentration of siRNA in transfections was 50 nM.

### Glioblastoma tumor growth in mice

Animal experiments in this study were approved by the Institutional Ethics Committee of West China Hospital of Sichuan University. Female BALB/c nude mice (4–6 weeks old) were obtained from GemPharmatech (Jiangsu, China) and randomly allocated to receive intracranial injection of U87-MG cells (3 × 10^5^ per animal) that had been transduced by recombinant lentivirus to overexpress IKIP or not. Cells were injected into the frontal region of the cerebral cortex at 2 mm lateral (right), 1 mm anterior and 3 mm ventral to bregma. At 30 days after injection, mice were euthanized and their brains removed, fixed in formalin, embedded in paraffin, and sectioned to a thickness of 4 μm.

### Immunohistochemistry

Immunohistochemistry of brain sections was performed as described [6]. Antigens were retrieved using citrate or EDTA. Sections were treated with hydrogen peroxide for 20 min to saturate endogenous peroxidases, and blocked for 1 h at room temperature using 5% bovine serum albumin. Sections were incubated with primary antibodies at 4°C overnight, followed by appropriate secondary antibody at room temperature for 1 h. Sections were treated with 3,3′-diaminobenzidine, followed by hematoxylin for counterstaining, and finally dehydrated using an ethanol gradient.

### Immunofluorescence

Brain sections were cut to a thickness of 4 μm, washed three times in phosphate-buffered saline (PBS), permeabilized for 10 min at room temperature with PBS containing 0.1%–0.3% Triton X-100, blocked for 1 h with 5% bovine serum albumin, washed with PBS, incubated at 4°C overnight with primary antibodies, then incubated at room temperature for 1 h with fluorescein-conjugated secondary antibody. Nuclei were counterstained with 4,6-diamidino-2-phenylindole (DAPI). Coverslips were mounted using antifade mounting medium. Images were acquired using a confocal microscope and fluorescence was quantified using ImageJ (US National Institutes of Health, Bethesda, MD, USA).

### Western blotting

Total protein was extracted from brain tissues or GBM cells using RIPA lysis buffer and quantified using the BCA protein assay. Equal amounts of protein were fractionated by SDS-PAGE and transferred to a polyvinylidene difluoride membrane, which was then blocked with blocking buffer (Epizyme, catalog no. PS108, Shanghai, China) for 20 min at room temperature, incubated at 4°C overnight with primary antibody, washed three times with TBS-T buffer, and incubated at room temperature for 1 h with secondary antibody. Antibody binding was visualized using enhanced chemiluminescence (SuperKine™ West Femto Maximum Sensitivity Substrate, Abbkine, catalog no. BMU102-CN, Wuhan, China) and quantified using ImageJ.

### Quantitative real-time PCR

Total RNA was extracted using Trizol reagent (R0016, Beyotime, Shanghai, China) according to the manufacturer’s protocol, converted into cDNA using a Hiscript II Q RT SuperMix kit, and quantified by quantitative PCR using the ChamQ Universal SYBR qPCR Master Mix kit. The following primers were used in the PCR: IKIP forward, GCAGGATGCTTTCAGTAGACTCA; IKIP reverse, GCAAAGGTGGAAAACCAATACCAG; THBS1 forward, AGACTCCGCATCGCAAAGG; THBS1 reverse, TCACCACGTTGTTGTCAAGGG; GAPDH forward, TCCCACCTTTCTCATCCAA; and GAPDH reverse, CCACATCACCCCTCTACCTC. Levels of target mRNAs were expressed relative to that of GAPDH mRNA using the 2^−ΔΔCt^ method.

### Cell viability

Viability of glioblastoma cells in culture was assessed using the Cell Counting Kit-8 (M4839, AbMole, USA). Cells were seeded into a 96-well plate in five replicate wells (2000 cells per well), then 10 μl CCK-8 solution was added to wells at each time point, the plate was incubated at 37°C for 2 h, and optical density at 450 nm was measured on a microplate reader.

### Cell proliferation

Proliferation of glioblastoma cells in culture was measured using the Incucyte Live-Cell Analysis System. Cells were seeded into a 96-well plate in five replicate wells (2000 cells per well) and imaged under a 10× objective every 8 h for up to 72 or 96 h. Relative confluence of cultures was calculated at each time point.

### Colony formation

Glioblastoma cells were seeded into 12-well plates (1000 cells per well), cultured for approximately 14 days, fixed with 4% paraformaldehyde for 20 min, and stained with 0.1% crystal violet for 20 min. Colonies were counted using ImageJ.

### Transwell assay of migration and invasion

In migration assays, 2 × 10^4^ glioblastoma cells in 200 μL of serum-free DMEM were added to the upper chamber of a transwell insert with 8-μm pore size in 24-well plates (catalog no. 353097, Corning). The same was done in invasion assays, except that the transwell filter was precoated with 60 μl of Matrigel (diluted 1:9 in DMEM). In both assays, 750 μL of DMEM containing 10% fetal bovine serum was added to the bottom chamber as a chemo-attractant.

Plates were incubated for 24 h at 37°C, after which cells were fixed with 4% paraformaldehyde for 20 min, then stained with 0.1% crystal violet for 20 min. The cells on the upper surface of membrane were scraped using a cotton ball, and the migrated or invaded cells were counted in five randomly selected visual fields per sample at 10× magnification under an inverted microscope.

### Wound healing assay of migration

Glioblastoma cells were seeded into 12-well plates (3 × 10^5^ cells per well) and incubated until confluence. The monolayer was scratched with a sterile disposable serological pipette (200 μL), then cultures were washed twice with PBS to remove detached cells and incubated for 24 h in serum-free DMEM. The width of the cell-free part of the scratched area was quantified immediately after the scratch and 24 h later at 10× magnification.

### Statistical analysis

All data were analyzed using GraphPad Prism 7.00 and reported as mean ± SD or mean ± SEM. Pairwise differences were assessed for significance using Student’s *t* test or the Mann-Whitney test. Differences were considered significant when *p* < 0.05.

## Results

### IKIP is upregulated in glioma tumors and higher expression is associated with worse prognosis

**Figure 1:**
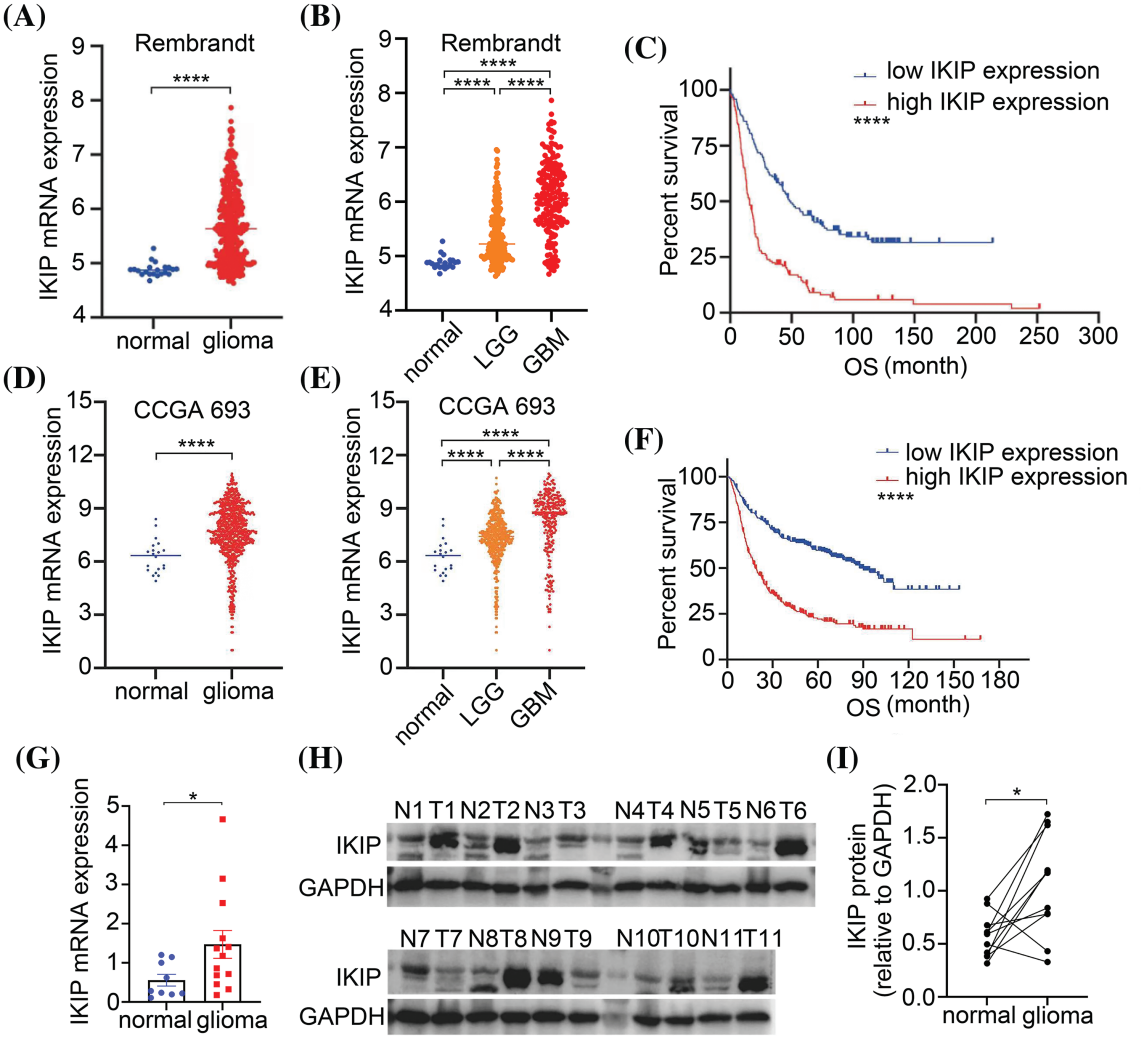
IKIP is upregulated in gliomas and its higher expression is associated with worse survival. (A) IKIP expression between normal and glioma samples in the Rembrandt database. (B) IKIP expression in normal, low-grade gliome (LGG) or glioblastoma (GBM) samples in the Rembrandt database. (C) Kaplan-Meier survival analysis of glioma patients based on IKIP expression in the Rembrandt database. (D) IKIP expression between normal and glioma samples in the Chinese Glioma Genome Atlas. (E) IKIP expression in normal, LGG and GBM samples in the Chinese Glioma Genome Atlas. (F) Kaplan-Meier survival analysis of glioma patients based on IKIP expression in the Chinese Glioma Genome Atlas. (G) Levels of IKIP mRNA in patient samples based on quantitative PCR. (H and I) Levels of IKIP protein in paired normal and glioma tissues from 11 patients based on western blotting. N, normal; T, tumor; OS, overall survival. *p < 0.05; ****p < 0.0001.

Levels of IKIP mRNA were significantly higher in low-grade glioma tissues and GBM tissues than in normal brain tissues in data from the public databases Rembrandt and the Chinese Glioma Genome Atlas (Figs. 1A, 1B). Kaplan-Meier analysis showed higher IKIP expression to be associated with worse prognosis based on data in the Rembrandt database (Fig. 1C) and the Chinese Glioma Genome Atlas (Figs. 1D–1F).

We confirmed the upregulation of IKIP in gliomas by comparing its mRNA levels between glioma tissues from 13 patients and normal brain tissues from another nine patients (Fig. 1G), and protein levels between paired tumor and adjacent normal tissues from another 11 patients (Figs. 1H, 1I).

### IKIP overexpression promotes proliferation of glioblastoma cells but inhibits their migration and invasion

**Figure 2:**
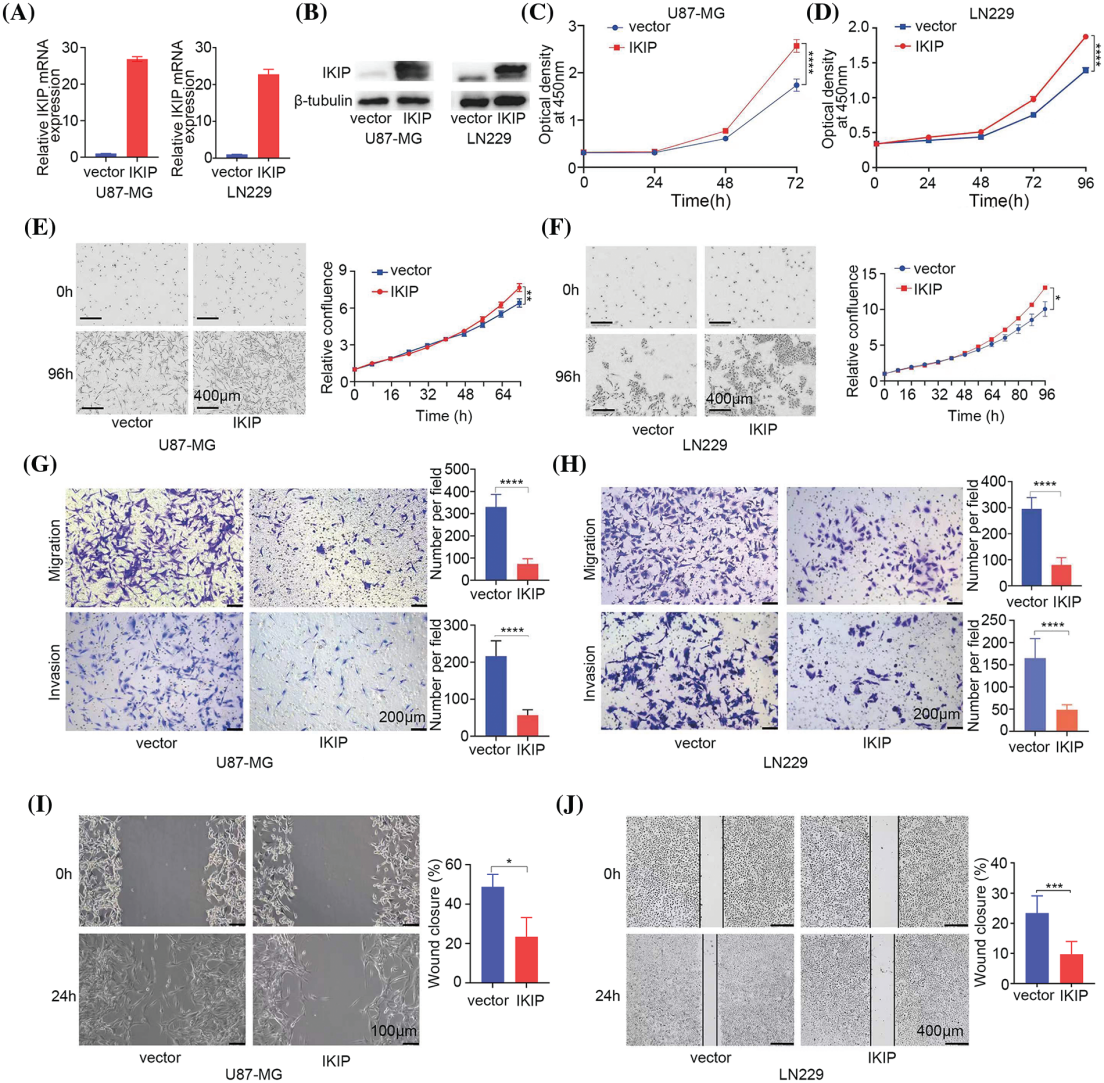
IKIP overexpression promotes proliferation of glioblastoma cells but inhibits their migration and invasion. (A and B) Levels of IKIP mRNA and protein in two glioblastoma cell lines after infection with lentivirus encoding human IKIP or no exogenous IKIP (“vector”). (C–F) Viability of the infected cells based on the (C and D) CCK-8 assay or (E and F) Incucyte assay. Scale bar in (E and F): 400 μm. (G and H) Transwell assays of migration and invasion by the infected cells. Scale bar: 200 μm. (I and J) Wound healing assays of migration by infected cells. Scale bar, U87-MG: 100 μm; LN229: 400 μm. *p < 0.05; **p < 0.01; ***p < 0.001; ****p < 0.0001.

We transduced the glioblastoma cell lines U87-MG and LN229 with lentivirus encoding human IKIP or not, and confirmed the corresponding overexpression or normal expression of IKIP using quantitative PCR and western blotting (Figs. 2A, 2B). IKIP overexpression increased the viability of both cell lines in the CCK-8 assay (Figs. 2C, 2D) and the Incucyte assay (Figs. 2E, 2F). At the same time, IKIP overexpression markedly inhibited migration and invasion of both cell lines in transwell assays (Figs. 2G, 2H) and wound healing assays (Figs. 2I, 2J).

### IKIP knockdown suppresses proliferation of glioblastoma cells but promotes their migration and invasion

**Figure 3:**
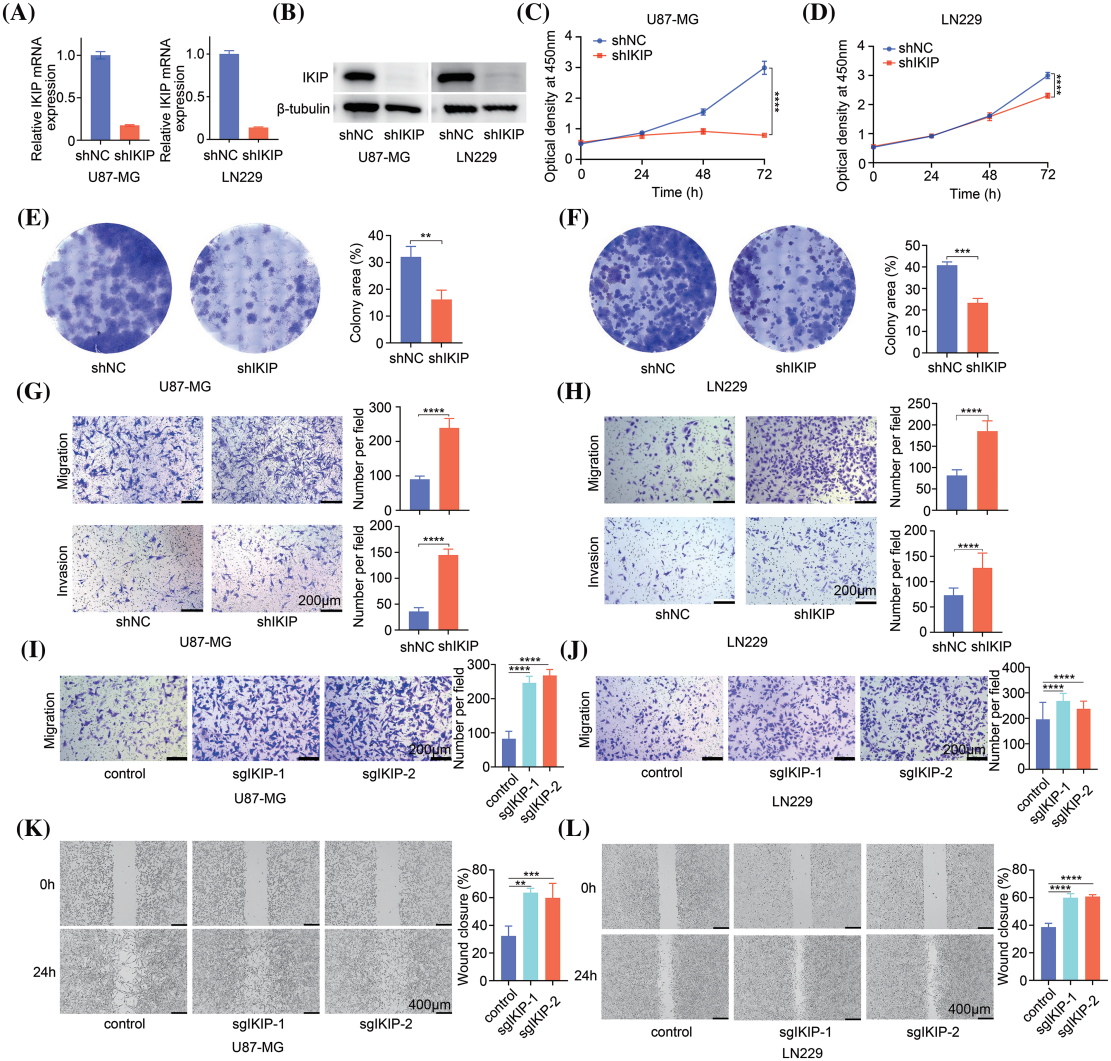
IKIP silencing inhibits proliferation of glioblastoma cells but promotes their migration and invasion. (A and B) Levels of IKIP mRNA and protein in two glioblastoma cell lines after transduction with lentivirus encoding short hairpin RNA targeting IKIP (“shIKIP”) or a scrambled negative-control RNA (“shNC”). (C and D) Viability of the transduced cells based on the CCK-8 assay. (E and F) Proliferation of the transduced cells based on the colony formation assay. (G and H) Transwell assays of migration and invasion by the transduced cells. Scale bar in (G and H): 200 μm. (I and J) Transwell assays of migration and invasion by cells transduced with lentivirus encoding one of two single-guide RNAs (sgIKIP-1, -2) to delete the IKIP gene via CRISP-Cas9. Scale bar in (I and J): 200 μm. (K and L) Wound healing assays of the cells described in panels I and J. Scale bar in (K and L): 400 μm. **p < 0.01; ***p < 0.001; ****p < 0.0001.

After observing effects of IKIP overexpression on migration and invasion of glioblastoma cells, we analyzed the effects of IKIP knockdown. The GBM cell lines U87-MG and LN229 were transduced with lentiviruses expressing a short hairpin RNA targeting IKIP or a scrambled negative-control RNA. We confirmed knockdown using quantitative PCR (Fig. 3A) and western blotting (Fig. 3B). Knockdown had the opposite effects on both cell lines as overexpression, inhibiting cell viability (Figs. 3C, 3D) and proliferation (Figs. 3E, 3F), but enhancing migration and invasion (Figs. 3G, 3H).

To corroborate these results using a different method to knock down IKIP, we transduced the two glioblastoma cell lines with lentivirus expressing single-guide RNAs to induce IKIP deletion via CRISPR-Cas9. We confirmed the knockout efficiency of sgRNAs using Western blot and DNA sequencing (Suppl. Fig. S1). Similar to our results with short hairpin RNA, CRISPR-mediated knockdown of IKIP enhanced migration and invasion by glioblastoma cells (Figs. 3I–3L).

### IKIP may reorganize the extracellular matrix in GBM

**Figure 4:**
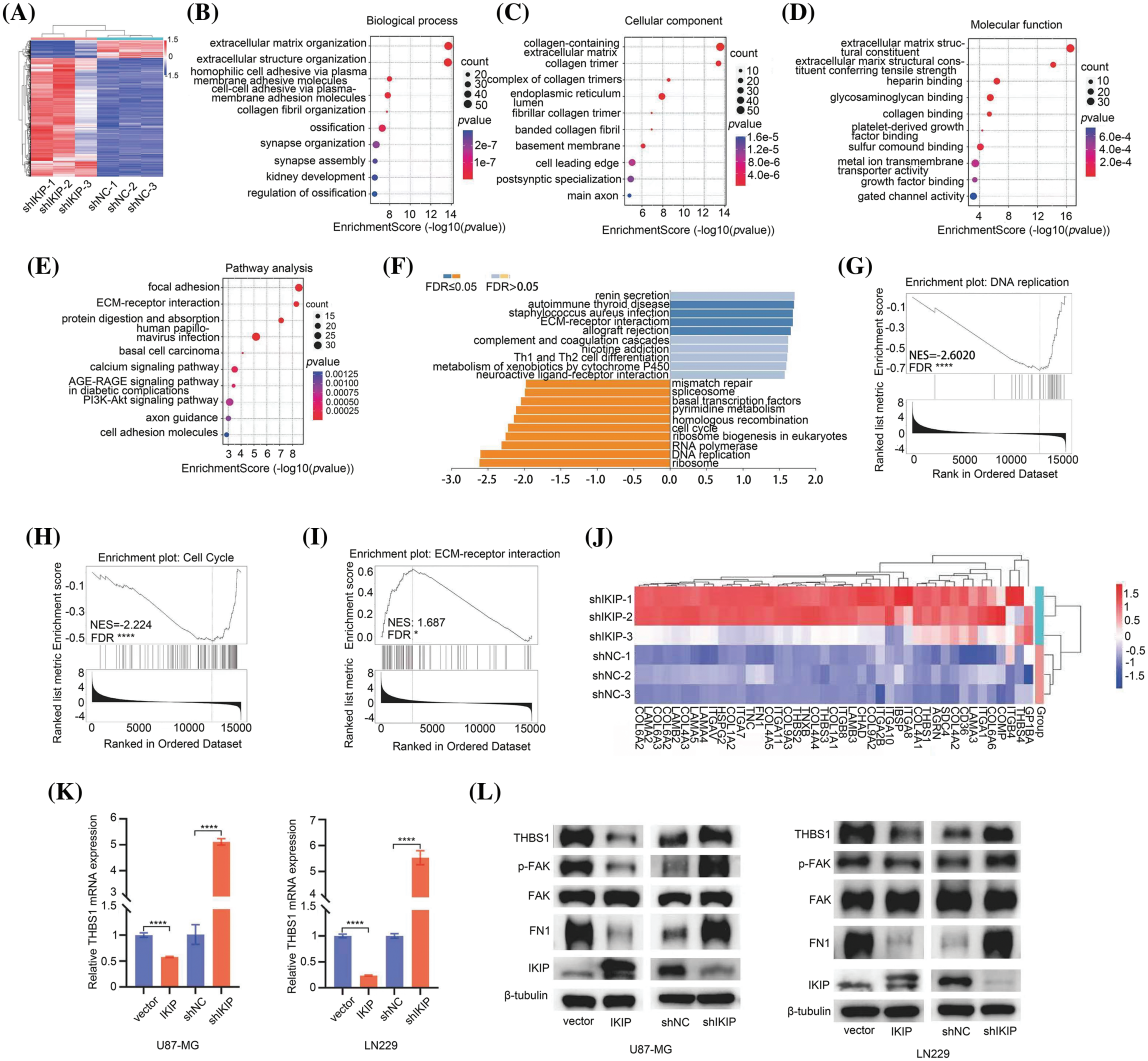
IKIP may reorganize the extracellular matrix of glioblastoma tumors by inhibiting THBS1/FAK signaling. Experiments involved the glioblastoma cell lines U87-MG or LN229 that had been transduced with lentivirus to overexpress IKIP (“IKIP”), a short hairpin RNA targeting IKIP (“shIKIP”), a negative-control RNA (“shNC”) or no transgene (“Vector”). (A) Heatmap of differential genes between U87-MG-shNC and U87-MG-shIKIP cells. Red indicates genes significantly upregulated in U87-MG-shIKIP cells; blue, genes significantly downregulated. (B–D) Enrichment of differentially expressed genes in (B) biological processes, (C) cell compartments and (D) molecular functions in the Gene Ontology taxonomy. Only the top 10 enriched terms are listed. (E) Enrichment of differentially expressed genes in Kyoto Encyclopedia of Genes and Genomes pathways. Only the top 10 enriched terms are listed. (F–I) Gene set enrichment analysis of genes differentially expressed between U87-MG-shNC and U87-MG-shIKIP cells, with detailed views of enrichment in (G) DNA replication, (H) cell cycle and (I) interactions between receptors and extracellular matrix. (J) Heatmap of the expression of selected genes involved in interactions between receptors and extracellular matrix in U87-MG-shNC and U87-MG-shIKIP cells. (K) Levels of THBS1 mRNA in U87-MG and LN229 cells, based on quantitative PCR. (L) Levels of THBS1, FAK, phospho-FAK, FN1 and IKIP in U87-MG and LN229 cells, based on western blotting. *p < 0.05; ****p < 0.0001.

In order to identify molecules and pathways that may explain these effects of IKIP on GBM cells, we compared the transcriptomes of U87-MG cells in which IKIP was knocked down with a short hairpin RNA or not. The analysis generated1406 genes that were upregulated and 207 that were downregulated following IKIP knockdown (Fig. 4A). The differentially expressed genes were enriched in Gene Ontology molecular functions related to cell adhesion and extracellular matrix (Figs. 4B–4D) and Kyoto Encyclopedia of Genes and Genomes (KEGG) pathways related to interactions between extracellular matrix and receptors and to cell adhesion to extracellular matrix (Fig. 4E). Gene set enrichment analysis showed that IKIP knockdown suppressed cell proliferation by inhibiting DNA replication and the cell cycle (Figs. 4G, 4H), while it promoted interactions between glioblastoma cell receptors and extracellular matrix (Figs. 4F, 4I, 4J). These findings are consistent with our observations that IKIP knockdown suppressed proliferation of glioblastoma cells but promoted their migration and invasion.

Next, we compared the transcriptomes of U87-MG cells in which IKIP was overexpressed or not, identifying 257 that were upregulated and 117 downregulated in response to overexpression (Suppl. Fig. S2A). The downregulated genes were enriched primarily in Gene Ontology terms involving the extracellular matrix, cell migration and metastasis (Suppl. Fig. S2B), and in KEGG pathways involving focal adhesion and interactions between receptors and extracellular matrix (Suppl. Fig. S2C).

### IKIP downregulates THBS1 and inhibits THBS1/FAK signaling

During analysis of the genes whose expression changed when IKIP was overexpressed or deleted, we found that THBS1 expression varied inversely with IKIP expression (Tables S1–S2). THBS1 is a homotrimeric glycoprotein that is secreted into the extracellular environment, where it helps construct and organize extracellular matrix [7–9]. It has previously been shown to contribute to invasiveness of glioblastoma cells [10–12]. Therefore, we hypothesized that the upregulation of IKIP in glioblastoma may downregulate THBS1 and thereby inhibit migration and invasion by tumor cells. We found higher THBS1 expression in GBM tissues than in normal brain tissues in the Cancer Genome Atlas (Suppl. Fig. S2D), and survival of glioma patients was worse when THBS1 expression was higher (Suppl. Fig. S2E). We confirmed these bioinformatic findings by showing that IKIP overexpression in the two glioblastoma cell lines LN229 and U87-MG sharply reduced THBS1 expression at the level of mRNA (Fig. 4K) and protein (Fig. 4L), whereas IKIP knockdown had the opposite effects.

We further verified these associations by showing that IKIP overexpression in these cultures inhibited the phosphorylation of focal adhesion kinase (FAK), a downstream target of THBS1 (Fig. 4L), while IKIP knockdown stimulated its phosphorylation. Activation of FAK through phosphorylation is associated with metastasis and poor prognosis in several cancers [13–15], and it promotes invasion and migration of glioblastoma cells [16]. Similar to its effects on THBS1, IKIP overexpression in the cultures downregulated fibronectin 1 (FN1), which participates in building up the entire ECM structure [17]. IKIP knockdown had the opposite effect.

These experiments suggested that IKIP regulates THBS1/FAK signaling in glioblastoma tumors, leading to reorganization of extracellular matrix that affects migration and invasion by tumor cells.

### THBS1 downregulation mimics the effects of IKIP upregulation in promoting migration and invasion by glioblastoma cells

**Figure 5:**
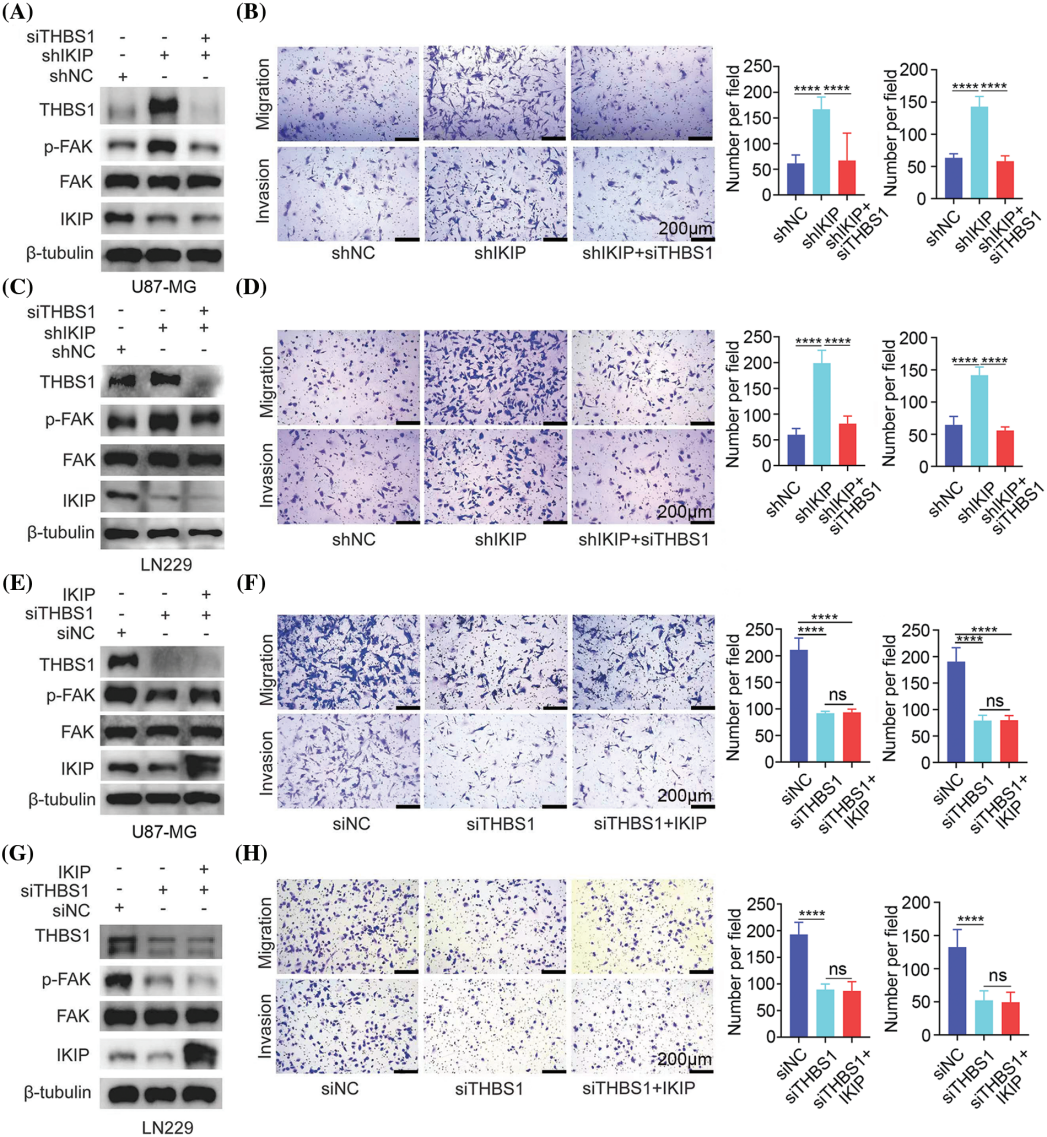
THBS1 downregulation mimics the effects of IKIP upregulation in promoting migration and invasion by glioblastoma cells. Experiments involved the glioblastoma cell lines U87-MG or LN229 that had been transduced with lentivirus to overexpress IKIP (“IKIP”), a short hairpin RNA targeting IKIP (“shIKIP”) or a negative-control RNA (“shNC”). Where indicated, cultures were transfected with small interfering RNA against THBS1 (“siTHBS1”) or a negative-control RNA (“siNC”). (A and B) Levels of protein in U87-MG cells in which IKIP was downregulated or not were examined using western blotting, while cell migration and invasion were analyzed in transwell assays. (C and D) The experiments in panels (A) and (B) were repeated with LN229 cells in which IKIP was downregulated or not. (E and F) The experiments in panels (A) and (B) were repeated with U87-MG cells in which IKIP was overexpressed or not. (G and H) The experiments in panels (A) and (B) were repeated with LN229 cells in which IKIP was overexpressed or not. Scale bar in this figure is 200 μm. ****p < 0.0001; NS, not significant.

To verify our hypothesis, we asked whether downregulating THBS1 would antagonize the ability of IKIP knockdown to promote migration and invasion by U87-MG and LN229 glioblastoma cells. Indeed, knocking down THBS1 using either of two small interfering RNAs (Suppl. Fig. S3) antagonized IKIP-mediated increases in levels of THBS1 and phosphorylated FAK (Figs. 5A–5C) and increases in migration and invasion (Figs. 5B–5D).

Next, we asked whether THBS1 is the only mediator, or potentially one of several mediators, of the effects of IKIP on migration and invasion by glioblastoma cells. We reasoned that if it is the only or primary mediator, knocking it down should maximally suppress FAK activation, migration and invasion—with no additional suppression when IKIP is upregulated. Indeed, we found that overexpressing IKIP in U87-MG or LN229 cells in which THBS1 was silenced did not suppress phosphorylation of FAK, migration or invasion more than THBS1 silencing on its own (Figs. 5E–5H). These results suggest that THBS1 is the primary downstream mediator of the effects of IKIP on migration and invasion by glioblastoma cells.

### IKIP promotes proliferation of glioblastoma tumors in mice but inhibits their invasion of surrounding tissues

**Figure 6:**
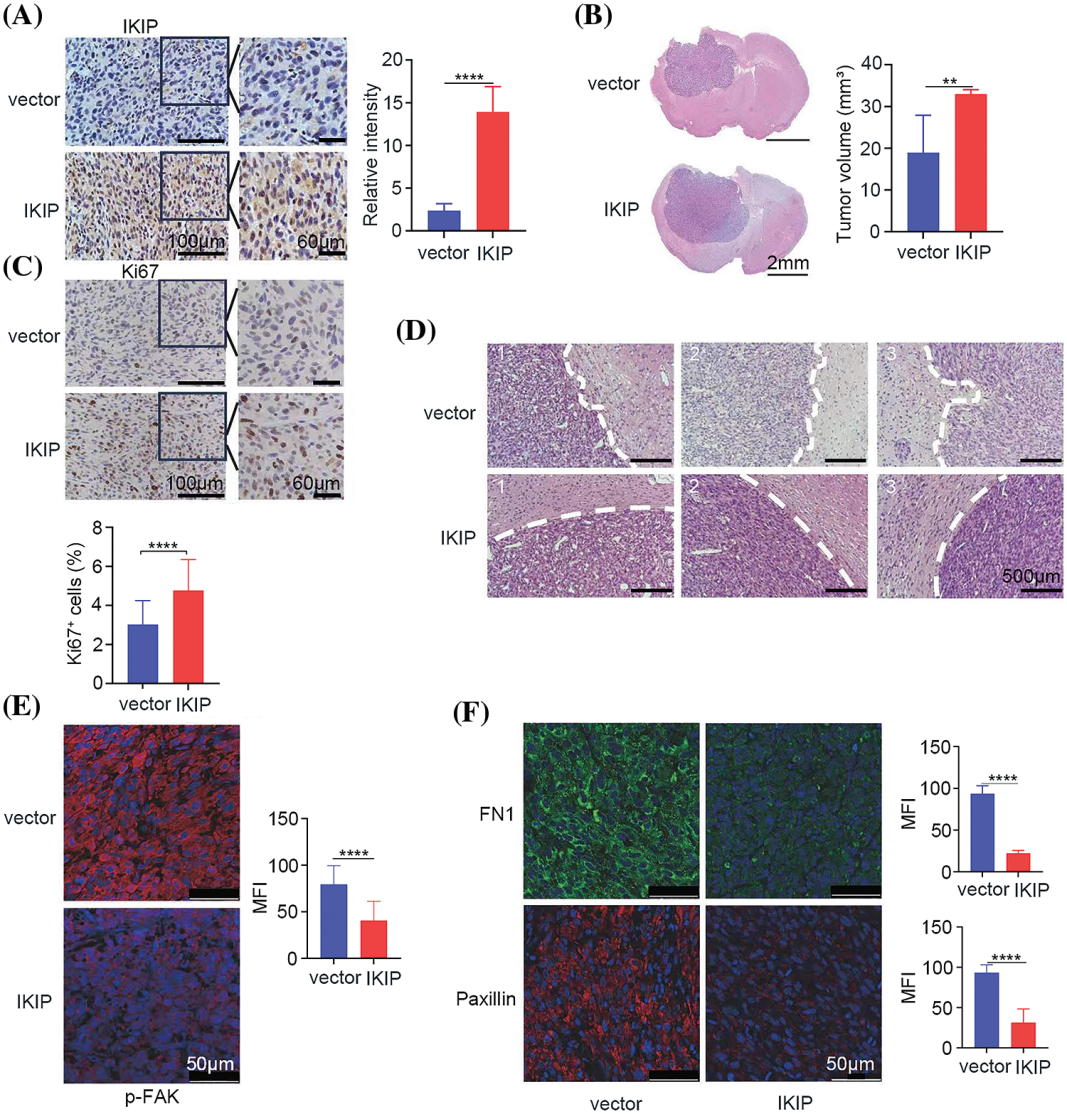
IKIP promotes growth of glioblastoma tumors in mice but inhibits their invasion into surrounding tissue. Animals were intracranially injected with U87-MG cells overexpressing IKIP (“IKIP”) or not (“vector”). Brain sections and tumor volume were analyzed at 30 days after tumor cell injection. (A) Brain sections were immunostained against IKIP. The region boxed in black is shown at higher magnification on the *right*. Scale bar in (A): 100 μm and 60 μm. (B) Brain sections were stained with hematoxylin-eosin to assess tumor volume. Scale bar in (B): 2 mm. (C) Brain sections were immunostained against Ki67. The region boxed in black is shown at higher magnification on the *right*. Scale bar in (C): 100 μm and 60 μm. (D) Brain sections were stained with hematoxylin-eosin and examined at higher magnification to assess tumor invasion into surrounding tissue. Scale bar in (D): 500 μm. (E and F) Brain sections were immunostained against phosphorylated FAK, FN1 and paxillin. Scale bar in (E and F): 50 μm. **p < 0.01; ****p < 0.0001; MFI, mean fluorescence intensity.

Finally, we complemented these *in vitro* studies by examining U87-MG tumors intracranially implanted into mice. The tumors had been transduced with recombinant lentivirus to overexpress IKIP or not, which we confirmed by examining tumors at 30 days after injection (Fig. 6A). IKIP overexpression led to larger tumors (Fig. 6B); higher proportion of Ki67-positive tumor cells (Fig. 6C), indicating greater proliferation; and reduced invasion into surrounding tissue, based on smoother tumor margin (Fig. 6D). These effects of IKIP overexpression were associated with reduced phosphorylation of FAK (Fig. 6E) as well as downregulation of FN1 and paxillin (Fig. 6F), which serve as markers of extracellular matrix. All these results corroborate our *in vitro* findings to suggest that IKIP suppresses invasion by glioblastoma tissues by reorganizing extracellular matrix.

## Discussion

In the present study, we focused on the role of IKIP in the migration and invasion of GBM cells. We validated that the mRNA and protein levels of IKIP are upregulated in glioma samples than that in the normal brain tissues. Our study provided evidence that IKIP suppresses the migration and invasion of GBM cells. Mechanistically, we found that IKIP downregulates the expression of THBS1 and thus restrains the THBS1/FAK signaling pathway. Although IKIP is highly expressed in glioma, it inhibits the migration and invasion of tumor cells by downregulating the THBS1/FAK signaling pathway.

A better understanding of the intrinsic and/or extrinsic molecular mechanisms that affect GBM cell migration may reveal opportunities for more effective therapy in inhibiting infiltration early in the disease course [18]. Compared to the wide exploration of the cellular and intracellular mediators of cytotoxicity, extracellular factors in the GBM tumor microenvironment that promote GBM invasion are under-studied. As a constitute of the tumor microenvironment, ECM consists of over 300 proteins and its remodeling has been demonstrated to favor tumor migration and metastasis [19,20]. The mechanical properties of the ECM have an impact on fibronectin fibril assembly, cytoskeletal stiffness, and strength of integrin-cytoskeleton linkages, the factors identified to be critical for cell motility [21]. On highly rigid ECMs, tumor cells spread and migrate rapidly by forming prominent stress fibers and mature focal adhesions [22]. THBS1 is an important molecule in the ECM that has been shown to promote tumor development in several cancers, including GBM [7,12,13,23–26]. De Fraipont et al. found that an N-terminal fragment of THBS1 enhances the migration of GBM cells [27]. Other studies also demonstrated that THBS1 and its downstream FAK/phospho-FAK signaling serve as the potential therapeutic target for GBM [28]. Here, we showed that IKIP participated in the remodeling of the ECM of GBM, inhibiting the focal adhesion of tumor cells and restraining GBM cell migration and invasion. Moreover, we further demonstrated that the THBS1/FAK signaling pathway mediated the inhibitory role of IKIP on GBM cell migration and invasion through rescue experiments. Therefore, our findings revealed that IKIP could suppress GBM migration and invasion through downregulating THBS1/FAK signaling, although IKIP is significantly upregulated in GBM and enhances cellular proliferation. This phenomenon is not rare in cancer research. For example, Sun et al. found that FBXO22 possesses both protumorigenic and antimetastatic roles in breast cancer progression [29]. Proto-oncogene MYC is overexpressed in 25% of human breast cancers and drives malignancy progression in experimental animals [30]. However, the migration and invasion of breast cancer are suppressed by MYC overexpression. In addition, the Ski-related novel protein N has been reported to promote mammalian tumorigenesis but inhibit endothelial-to-mesenchymal transition (EMT) and tumor metastasis [31]. It has been proposed that the cancer can modulate the expression of genes depending on the progression stage. For instance, FBXO22 may help to establish the primary tumor colonies at the initial stages of tumorigenesis by increasing tumor proliferation and growth. Nevertheless, FBXO22 expression may be suppressed at the metastatic stage to allow tumor cells to undergo EMT and metastasize. Therefore, the authors suggested that the direct targeting of FBXO22 may enhance the metastatic capacity of breast cancer cells. In our study, IKIP is highly expressed in bulk glioma samples, but the spatial expression of IKIP is not known. The public database IVY GLIOBLASTOMA ATLAS PROJECT harbors the spatial mRNA expression information of GBM. Surprisingly, the expression level of IKIP was lower in the leading edge and infiltrating tumor compared to tumor zones with hyperplastic vessels and microvascular proliferation (Suppl. Fig. S4). Therefore, it seems reasonable that IKIP harbors both pro-proliferative and anti-metastatic roles in GBM.

IKIP is first reported to promote apoptosis in endothelial cells and negatively regulate inflammation [32–34]. Recently, the role of IKIP in GBM was revealed. Li et al. reported that IKIP is highly expressed in GBM compared to normal brain tissues or low-grade gliomas [4]. Functionally, they found that IKIP promotes cell cycle progression in GBM cells by inhibiting the ubiquitination and degradation of CDK4. In our study, we obtained the consistent result that IKIP promotes cell proliferation in GBM. In the study by Chen et al., the transient knockdown of IKIP inhibits the migration of the U87-MG cell line [5]. However, we did not obtain the same result using the GBM lines which stably expressed IKIP or shIKIP. We neither obtain the same result using the sgRNA-mediated knockout system. On the contrary, we found that IKIP overexpression markedly inhibits the migration and invasion of both U87-MG and LN229 cell lines. Moreover, shRNA- or sgRNA-mediated IKIP silencing promotes the migration and invasion of GBM cell lines. We also performed transcriptome analysis to verify our findings. The results suggested that IKIP knockdown leads to a positive regulation of tumor ECM, including the enriched terms “extracellular matrix organization”, “extracellular matrix”, and “collagen-containing extracellular matrix”. In addition, we again performed transcriptome analysis using U87-MG-vector and U87-MG-IKIP cell lines. Consistent with the results of the IKIP-knockdown program, we found that the genes downregulated upon IKIP overexpression are enriched in ECM and focal adhesion. Taken together, we found that IKIP suppresses the migration and invasion of GBM cell lines through remodeling the ECM. About the contradictory results between the present study and the previous report, we thought that it may be attributed to the different expression systems, namely the lentiviruses and transient siRNAs. Thus, future studies should be conducted to solve this controversy.

## Conclusions

In conclusion, our study provides evidence that IKIP downregulates the THBS1/FAK signaling pathway and remodels tumor ECM, inhibiting the migration and invasion of GBM. We suggest that IKIP has a dual role in GBM progression. Our research improves the comprehension of IKIP involved in GBM progression.

## Supplementary Materials

Figure S1 Validation of the IKIP knockout in U87-MG and LN229 cell lines. (A-B) Confirmation of IKIP knockdown in U87-MG and LN229 glioblastoma cell lines transduced with lentivirus encoding single-guide RNAs targeting IKIP (sgIKIP-1, -2). (C-D) Sequencing of PCR products of genomic DNAs of IKIP knocked out in U87-MG and LN229 cells by IKIP sgRNAs, respectively.

Figure S2 IKIP overexpression alters the transcriptome of glioblastoma cell line U87-MG. Cells were transduced with recombinant lentivirus encoding IKIP or not, then their transcriptomes were compared. (A) Volcano plot of genes differentially expressed between the two cell types. (B) Top 20 Gene Ontology terms enriched in genes that were downregulated when IKIP was overexpressed. (C) Top 10 Kyoto Encyclopedia of Genes and Genomes pathways enriched in genes that were downregulated when IKIP was overexpressed. (D) Comparison of THBS1 expression between glioblastoma tissues and normal brain tissues in the GEPIA database. (E) Comparison of overall survival between GBM patients in the Cancer Genome Atlas whose tumors showed low or high THBS1 expression. *p<0.05.

Figure S3 Confirmation of THBS1 knockdown at the level of (A) mRNA and (B) protein in U87-MG and LN229 glioblastoma cell lines transfected with small interfering RNA targeting THBS1 (siTHBS1-1, -2). The effect of knockdown on phosphorylation of FAK was also examined.

Figure S4 Comparison of IKIP expression in different zones of glioblastoma tumors. Data were taken from the Ivy Glioblastoma Atlas (https://glioblastoma.alleninstitute.org). ****p<0.0001.

## Data Availability

The data and other supporting materials used and/or analyzed in the current study are available from the corresponding authors.

## Abbreviations

IKIP: Inhibitor of NF-κB kinase-interacting protein
GBM: Glioblastoma
THBS1: Thrombospondin 1
ECM: Extracellular matrix
CGGA: Chinese Glioma Genome Atlas
TCGA: The Cancer Genome Atlas
IHC: Immunohistochemistry
IF: Immunofluorescence
SgRNA: Single-guide RNA
qPCR: Quantitative real-time PCR analysis
PVDF: Polyvinylidene difluoride
CCK-8: Cell Counting Kit-8
SD: Standard Deviation
SEM: Standard Error of the Mean
DEG: Differentially expressed gene
GSEA: Gene Set Enrichment Analysis
GO: Gene Ontology
BP: Biological process
CC: Cellular component
MF: Molecular function
KEGG: Kyoto Encyclopedia of Genes and Genomes

## Supplementary Materials

The supplementary material is available online at https://doi.org/10.32604/or.2024.042456.
