# Supplementary figures and images for: IKIP downregulates THBS1/FAK signaling to suppress migration and invasion by glioblastoma cells
Source: Oncol Res. 2024 Jun 20;32(7):1173–84. doi: 10.32604/or.2024.042456 (PMC11211642; doi:10.32604/or.2024.042456)

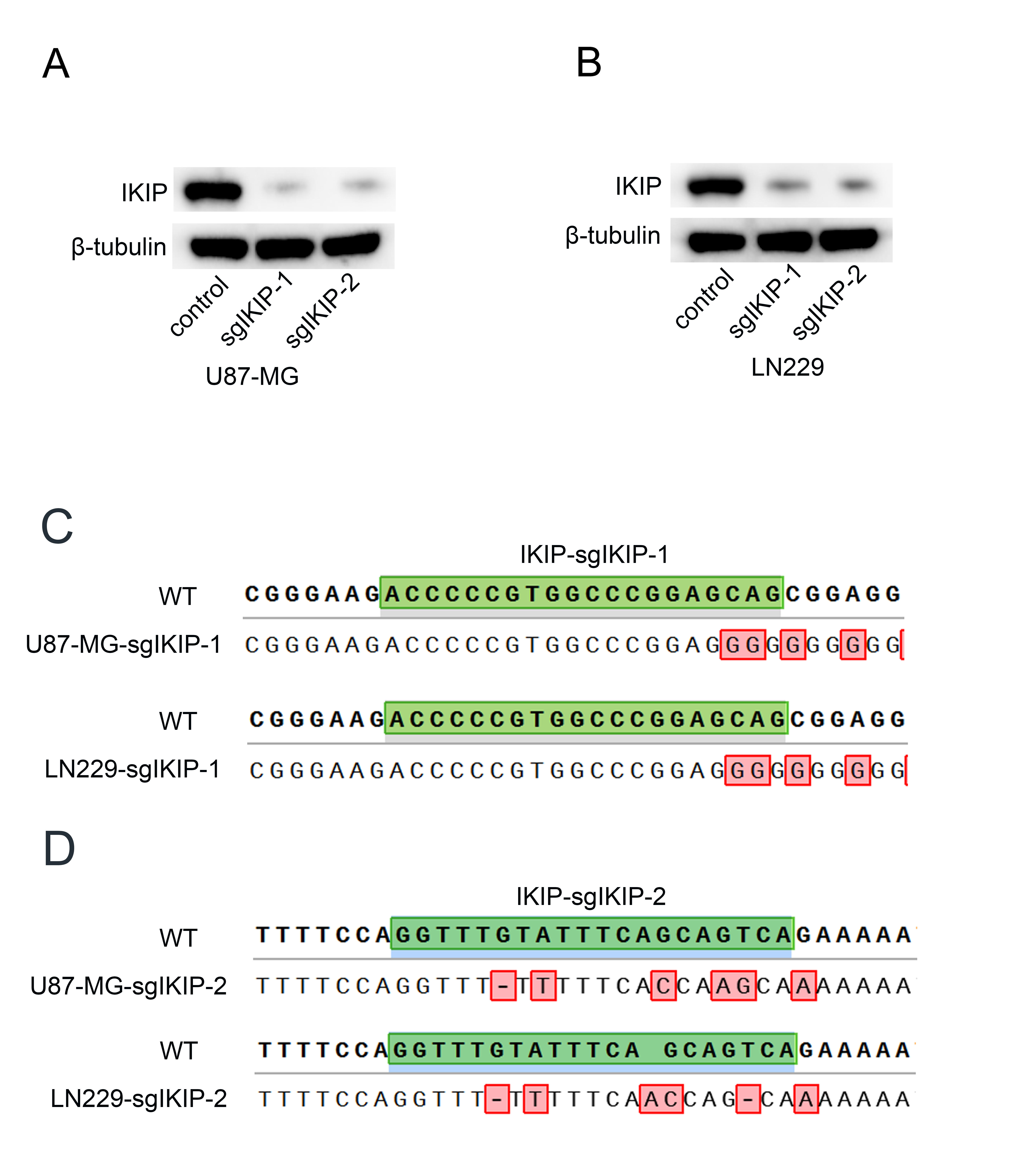

Supplement: Figure S1 [file OncolRes-32-42456-s001.tif]

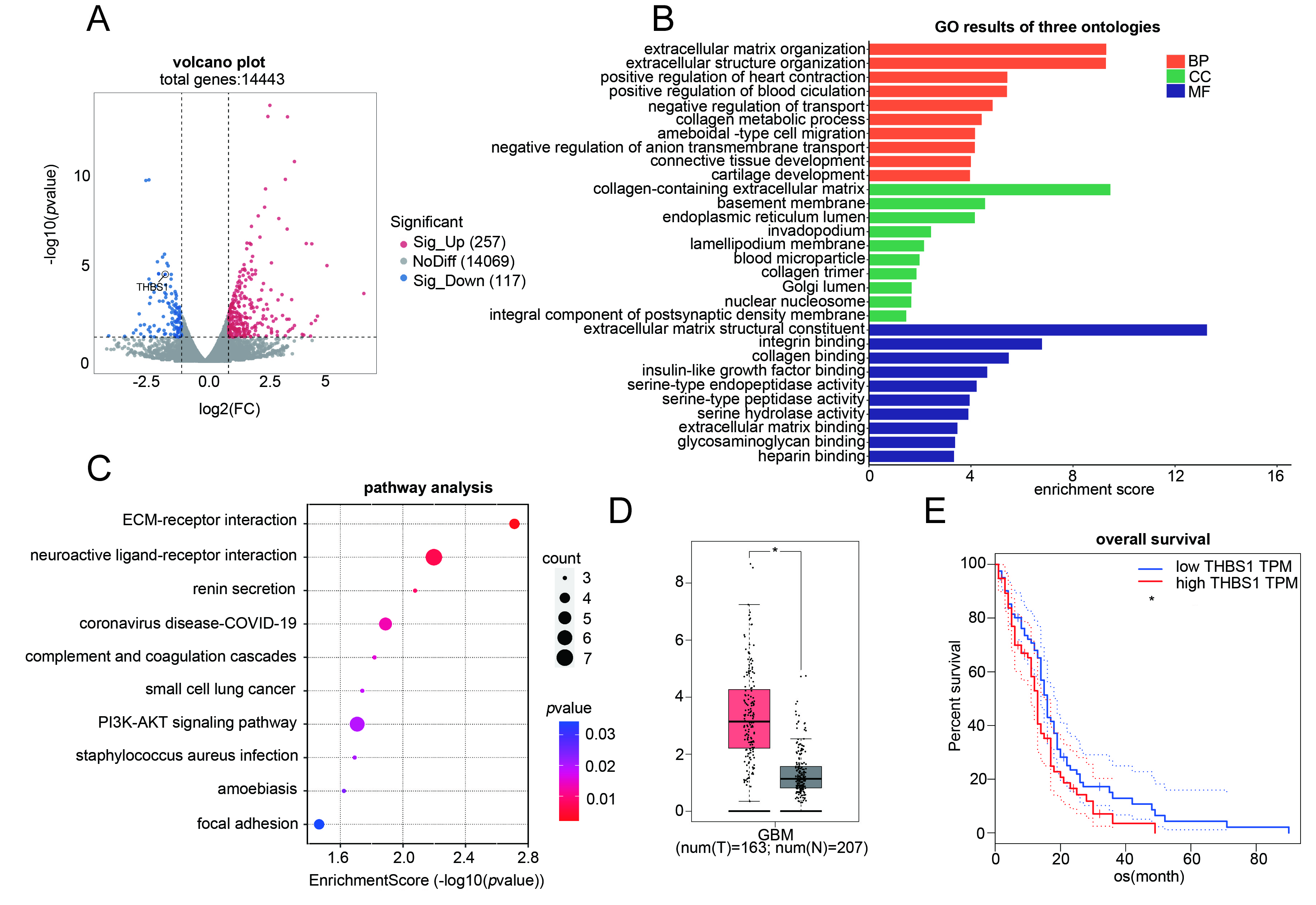

Supplement: Figure S2 [file OncolRes-32-42456-s002.tif]

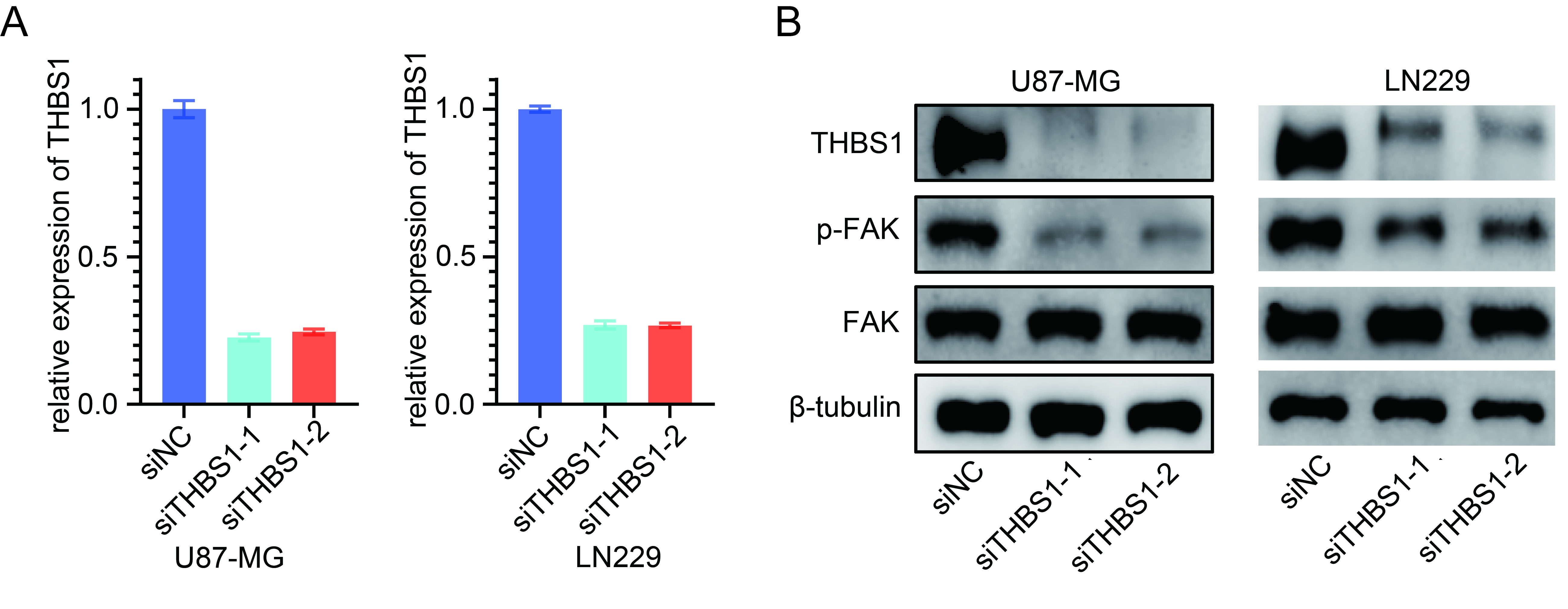

Supplement: Figure S3 [file OncolRes-32-42456-s003.tif]

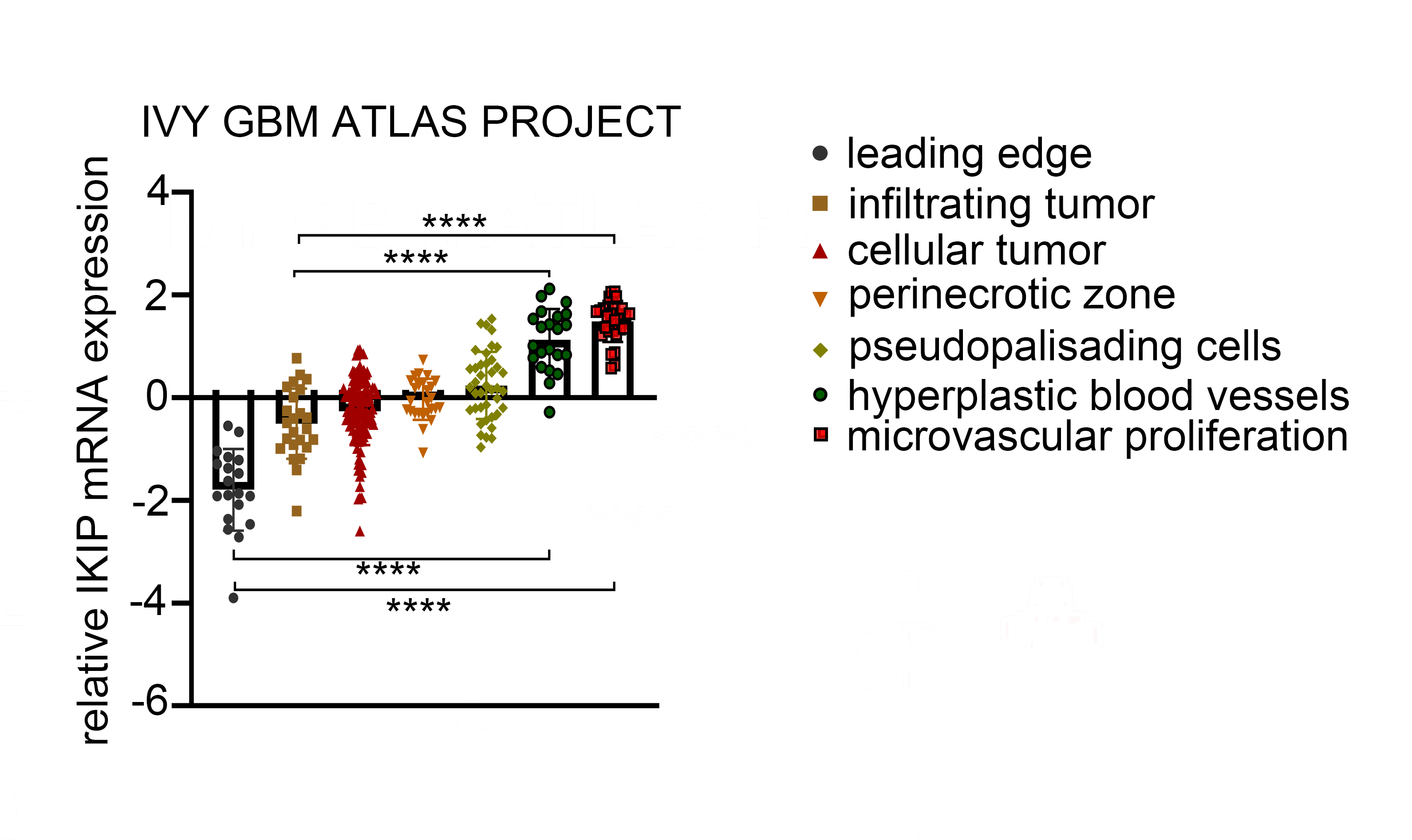

Supplement: Figure S4 [file OncolRes-32-42456-s004.tif]
